# Effects of Transglutaminase on the Protein Network and In Vitro Starch Digestibility of Asian Wheat Noodles

**DOI:** 10.3390/foods8120607

**Published:** 2019-11-22

**Authors:** May Sui Mei Wee, Christiani Jeyakumar Henry

**Affiliations:** 1 Clinical Nutrition Research Centre (CNRC), Singapore Institute for Clinical Sciences, Agency for Science, Technology and Research (A*STAR), Singapore 117599, Singapore; may_wee@sics.a-star.edu.sg; 2 Department of Biochemistry, Yong Loo Lin School of Medicine, National University of Singapore, Singapore 117599, Singapore

**Keywords:** transglutaminase, wheat noodles, starch digestibility, glycaemia, in-vitro digestion

## Abstract

Wheat noodles are a staple commonly consumed in Asia, but high intakes have been associated with type 2 diabetes due to its rapid starch digestibility. We hypothesised that protein network-binding via transglutaminase (TG) would form a stronger barrier encapsulating the starch granules to limit enzymatic access and digestion. The amount of glucose release decreased significantly with increasing TG concentration, with a reduction of approximately 16% with 2% TG after 120 min of digestion. The slower rate of glucose release during the first 60 min of digestion for 2% compared to 0% TG suggested impeded first stage enzymatic access rather than second stage starch hydrolysis into glucose. Upon increasing the TG concentration, confocal microscopy revealed a denser protein network with increased connectivity, supported by a decrease in protein solubility and gelatinisation enthalpy, and increased firmness and work of shear. Therefore, transglutaminase can potentially be used to reduce starch digestibility in wheat noodles via protein network-binding.

## 1. Introduction

Wheat noodles, such as *mee sua* (a type of white salted noodles), are a carbohydrate staple frequently consumed in Asia. However, high intakes of noodles and rice have been associated with metabolic disorders, in particular type 2 diabetes and obesity [1] due to their high carbohydrate content and its contribution to postprandial glycaemic response. These noodles are rapidly digested, which leads to a spike in postprandial glycaemic response, and chronic elevated blood glucose levels are a precursor for type 2 diabetes [2]. Asian wheat noodles have been reported to have a high glycaemic index * (GI) of 82 (glucose as a reference GI of 100) [3]. On the contrary, pasta (such as spaghetti), the Western counterpart, has a low GI ranging from between 32 to 58 [4]. 

The discrepancy in glycaemic impact between Asian wheat noodles and pasta may be due to several factors, such as ingredients and processing methods used. From a food structure perspective, its low digestibility is often attributed to the protein (gluten) matrix surrounding and encapsulating the starch granules, which prevents enzymatic access for digestion [5,6,7,8,9]. In some cases, proteins may also interact with starch granules at the surface, which could affect both enzymatic access as well as the subsequent rate of starch hydrolysis into glucose [10,11]. While Asian wheat noodles also contain gluten, the use of eggs and durum wheat (higher protein content), or processing methods such as drying [8,12] and extrusion [13] in pasta further modifies the protein network and starch digestibility. Therefore, how can the protein network of Asian noodles be modified to reduce its digestibility? Several strategies have been employed to reduce the digestibility of noodles and pasta, such as (1) using alternative high protein legume flours, such as chickpea [14,15]; (2) adding denatured protein to increase starch–protein interactions and limit the extent of starch gelatinisation [16]; or (3) drying at high temperatures to denature and bind the protein network [12]. In this study, we explore the use of a protein crosslinking agent, transglutaminase, to modify the protein network of Asian wheat noodles. 

Transglutaminase is an enzyme which forms covalent crosslinks between the proteins glutenin and gliadin, which are present in wheat flour for making noodles. Specifically, it catalyses the reaction between the amine (NH_2_) groups of glutamine and lysine to form covalent ε-(γ-glutamyl)lysine bridges (G-L bonds), which are not affected by processing (cooking) or the food matrix environment [17]. Traditionally, transglutaminase has been used in meat processing, such as for joining different meat cuts or increasing the elasticity of surimi products [18]. In noodles, transglutaminase has been used to improve the texture and storage quality of noodles, via increasing the firmness and elasticity of the noodles and reducing retrogradation effects [19]. Consequently, crosslinking of the gluten network may also inadvertently change its digestibility due to a stronger, denser protein network further limiting enzymatic access. The effect of transglutaminase specifically on starch digestibility has not been widely explored, and there is an opportunity to study its effects on the in vitro and in vivo digestibility of the noodles.

The objectives of this study were to determine the effects of transglutaminase treatment on (1) in vitro starch digestibility of wheat noodles, and (2) the protein network integrity in relation to starch based on microstructure, protein solubility, starch gelatinisation behaviour and noodle bulk properties (i.e., texture and water absorption). 

* Glycaemic Index (GI): The glycaemic index is the glycaemic response elicited by a portion of food containing 50 g of available carbohydrate. It is expressed as a percentage of the glycaemic response elicited by 50 g of the reference carbohydrate, which is either a glucose solution or white wheat bread.

## 2. Materials and Methods

### 2.1. Noodles Preparation

Noodle samples at different transglutaminase concentrations were produced using plain flour (200 g; 71.1% carbohydrate, 11.0% protein, 1.2% fat; Prima Flour, Singapore), water (75 g or 37.5% flour weight), salt (2 g or 1% flour weight), and transglutaminase enzyme powder (0–4 g or 0–2% flour weight; Activa™ UltraDente STG-M; Ajinomoto Co., Tokyo, Japan). Flour and salt were dry-blended before being added to the mixing compartment of an automated noodle maker (HR2365 Philips, Singapore). Transglutaminase powder was first dissolved and mixed in the water before being added to the dry ingredients, kneaded into a noodle dough for 8 min (setting 4) and then extruded (1 mm). The noodles were left to rest for 2 h at room temperature (20 °C) for the transglutaminase crosslinking action to take place before being stored at 4 °C prior to analysis. For cooked noodles, raw noodles (50 g portions) were weighed and cooked in boiling water (3 L) for 5 min, cooled immediately in cold water (1 L) for 1 min to stop further cooking, rinsed under running water for 1 min to remove excess starch, and then tossed 30 times in a strainer to remove excess water. All cooked samples were packed in airtight containers and stored at 4 °C overnight to rest prior to analysis.

### 2.2. Confocal Scanning Laser Microscopy (CSLM)

The microstructure of raw noodles was studied using confocal scanning laser microscopy (CSLM). Raw noodles were sliced using a cryostat (CM3050 S, Leica, Cryostat, Nussloch, Germany) to 50 μm thickness at −20 °C and mounted on glass slides. The sliced noodle sections were stained with 10 μL of a 0.25% *w*/*v* fluorescein isothiocyanate (FITC; Sigma-Aldrich, Saint Louis, MO, USA) and 0.025% *w*/*v* Rhodamine B (RB; Sigma-Aldrich, Saint Louis, MO, USA) mixture for a minimum of 10 min and covered with a coverslip [20]. The stained samples were observed using a confocal scanning laser microscope (FluoView 3000 Inverted, Olympus, Allentown, PA, USA) at excitation wavelengths of 488 and 561 nm for FITC and RB, respectively, at 20× magnification. Starch granules were stained green by FITC and protein was stained red/orange/yellow by RB, depending on the degree of co-localisation. A minimum of six slices for each sample imaged at various X-Y positions (fixed Z-depth) were taken to capture a representative image.

### 2.3. Protein Solubility

The protein solubilities of raw and cooked noodles in different extracting reagents were measured based on published methods [21,22]. Phosphate buffer (0.1 M; pH 6.8; PB) was prepared using sodium phosphate dibasic heptahydrate (0.0578 M; Sigma-Aldrich, Saint Louis, MO, USA) and sodium phosphate monobasic monohydrate (0.0422 M; Sigma-Aldrich, Saint Louis, MO, USA) in deionised (DI) water. Other extracting reagents were prepared by dissolving either urea (8 M; PB + Urea; Sigma-Aldrich, Saint Louis, MO, USA) or DL-dithiothreitol (0.05 M; PB + DTT; Sigma-Aldrich, Saint Louis, MO, USA) in 0.1 M phosphate buffer. All reagents were prepared fresh on the day of analysis. Raw and cooked noodle samples were weighed (0.1 g) and extracted with 1 mL of extracting reagent in a 2 mL microcentrifuge tube. The mixture was ultrasonicated (FB15055, Fisherbrand, Leicestershire, UK) for 60 min and maintained at temperatures below 30 °C. After extraction, the tubes were centrifuged at 10,000× *g* for 20 min at 20 °C. The amount of protein solubilised was quantified using the Bradford protein assay by adding the supernatant (0.1 mL) to Bradford reagent (0.1 mL; Sigma-Aldrich, Saint Louis, MO, USA), vortexed and transferred to microplates within 15 min to read the absorbance at 595 nm (Cytation 5, BioTek, Winooski, VT, USA). The standard absorbance curve was constructed using bovine serum albumin (BSA; Sigma-Aldrich, Saint Louis, MO, USA). All measurements for protein solubility were done in duplicates. The extracting reagents phosphate buffer (PB), phosphate buffer with 8 M urea (PB + Urea) and phosphate buffer with 0.05 M DL-dithiothreitol (PB + DTT) were used to disrupt free proteins, non-covalent hydrogen bonding and covalent disulphide linkages, respectively.

### 2.4. Differential Scanning Calorimetry (DSC)

Thermal properties of raw noodles were analysed using a differential scanning calorimeter (DSC; Netzsch DSC 214 Polyma) based on published methods [23]. Raw noodles were freeze-dried (Virtis SP Scientific, Warminster, PA, USA) for 48 hours and ground with a mortar and pestle. Ground samples (4–6 mg) were placed in Concavus aluminium pans (Netzsch, Germany) with 10 μL of DI water added. The pans were then hermetically sealed with lids. The analysis was carried out from 10 to 95 °C at a heating rate of 10 °C/min with an empty sealed reference pan. The parameters onset temperature (T_o_), peak temperature (T_p_), end temperature (T_e_) and enthalpy of gelatinisation (peak area; ΔH) were analysed using the Netzsch Proteus Thermal Analysis software (Netzsch, Germany). All measurements for DSC were done in triplicates.

### 2.5. Water Absorption 

Noodles (50 g raw noodle portions) were weighed before and after cooking to calculate water absorption (%) using equation (1). All measurements for water absorption were done in triplicates.

(1) $$\mathrm{Water}\;\mathrm{absorption}\;\left(\%\right)=\frac{\mathrm{Weight}\;\mathrm{of}\;\mathrm{cooked}\;\mathrm{noodles}-\mathrm{weight}\;\mathrm{of}\;\mathrm{raw}\;\mathrm{noodles}}{\mathrm{Weight}\;\mathrm{of}\;\mathrm{raw}\;\mathrm{noodles}}\times 100\%$$

### 2.6. Texture Analysis

Textural properties of cooked noodles (i.e., firmness (kg) and work of shear (kg·sec)) were measured using a texture analyser (TA.XT plus, Stable Microsystems Ltd., Surrey, England) and analysed using the Texture Expert program (version 6.1). Cooked noodles (approximately 80 g) were placed in a 600 mL beaker and compressed for a distance of 34.5 mm at a test speed of 0.5 mm/s using the Triple Ring Cutting (TRC) system probe and a 30-kg load cell. Samples were freshly cooked and analysed immediately prior to each compression test. Firmness (kg) is defined as the peak compression force, and work of shear (kg·sec) is calculated as the area under the force-time curves. All measurements for texture analysis were done in triplicates.

### 2.7. In Vitro Digestion 

The in vitro digestion protocol was adapted and modified from the INFOGEST digestion method [24]. Briefly, cooked noodles were subjected to oral (100 s), gastric (1 h) and intestinal (2 h) phase digestions and the total glucose released at the end was quantified using a colorimetric reducing sugar assay with dinitrosalicylic acid (DNS). The cooked noodles (5 g) were finely chopped into 5-mm pieces to standardise surface area and volume in duplicates. For the oral phase of digestion, oral phase solution (5 mL) consisting of α-amylase (12.5 mg/mL in simulated salivary fluid (SSF; pH 7.0); 4 mL; Sigma-Aldrich, USA) and 7.5 mM CaCl_2_ (1 mL; Sigma-Aldrich, Saint Louis, MO, USA) was added to the noodles, vortexed, and digested at 37 °C for 1 min 40 s. Gastric phase solution (10 mL) consisting of pepsin (9.94 mg/mL in simulated gastric fluid (SGF; pH 3.04); 8 mL; Sigma-Aldrich, Saint Louis, MO, USA), 0.3 M CaCl_2_ (5 μL), DI water (1.445 mL) and 1 M HCl (0.55 mL) was added to the noodles and vortexed after oral phase digestion for 1 h at 37 °C. After gastric phase digestion, the intestinal phase solution (20 mL) consisting of pancreatin (2.585 mg/mL in simulated intestinal fluid (SIF; pH 7.0); 16 mL; Sigma-Aldrich, Saint Louis, MO, USA), amyloglucosidase (80 μL; Sigma-Aldrich, Saint Louis, MO, USA), 0.3M CaCl_2_ (40 μL), DI water (3.48mL) and 1M NaOH (400 μL) was added to the gastric chyme and transferred into dialysis tubes (MWCO 6–8 kDa; Repligen, Boston, MA, USA) in 150 mL of SIF as the dialysate. The mixture was further digested for 2 h under constant stirring of the dialysate at 150 rpm and 37 °C. Aliquots (0.5 mL) of the dialysate from the intestinal phase digestion were taken out at 0, 5, 10, 15, 30, 60, 90 and 120 min intervals, and SIF (0.5 mL) was added back to the digesta to replace the withdrawn aliquot at each time point. The dialysate (0.1 mL) was added to 0.1 mL of DNS reagent (Sigma-Aldrich, Saint Louis, MO, USA), vortexed and placed in a boiling water bath (100 °C) for 10 min and left to cool for another 10 min. The mixture was further diluted with DI water (0.2 mL), transferred to a 96-well microplate and the absorbance was read at 540 nm (Cytation 5, BioTek, Winooski, VT, USA). The total amount of glucose released per gram of carbohydrate in noodles was quantified using a standard D-glucose (Sigma-Aldrich, Saint Louis, MO, USA) curve.

### 2.8. Statistical and Mathematical Analysis

Statistical analysis was performed using the paired *t*-test for comparing means of samples to control and between samples of different transglutaminase concentrations at 95% significance level (α = 0.05). Differentiation (first order derivative) analysis on glucose released with digestion time was performed using a mathematical graphing software, OriginPro (OriginPro, Version 2019b, OriginLab Corporation, Northhampton, MA, USA).

## 3. Results

### 3.1. Microstructure (CSLM)

**Figure 1:**
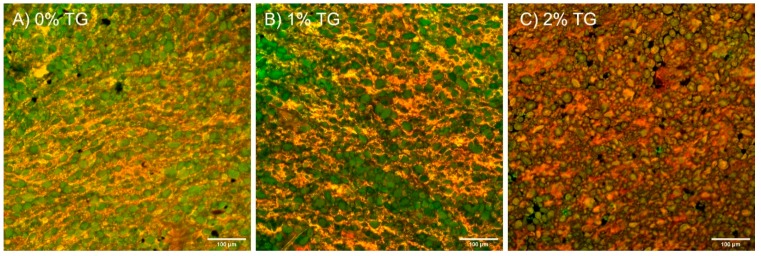
Microstructure of raw noodles with (**A**) 0%, (**B**) 1% and (**C**) 2% transglutaminase at 20 × magnification; scale bar represents 100 μm; green areas represent starch granules stained by 0.25% *w*/*v* fluorescein isothiocyanate (FITC) (488 nm) and yellow/orange areas represent protein stained by 0.025% *w*/*v* Rhodamine B (561 nm).

Without TG treatment (Figure 1A), the starch granules appeared to be more discrete and were more uniformly distributed within the protein matrix. The starch granules were also somewhat in laminar arrangement, which may have been in the direction of noodle extrusion, with a thin protein matrix layer alongside. With increasing TG concentration, the thickness of the protein network and contact with starch granules increased (Figure 1B,C). At 1% TG (Figure 1B), larger localised areas (orange) of the protein fraction were observed along with laminar regions (yellow). At 2% TG (Figure 1C), the laminar arrangement of starch granules was no longer distinctive, while localised areas of proteinaceous regions grew larger.

### 3.2. Protein Solubility

**Figure 2:**
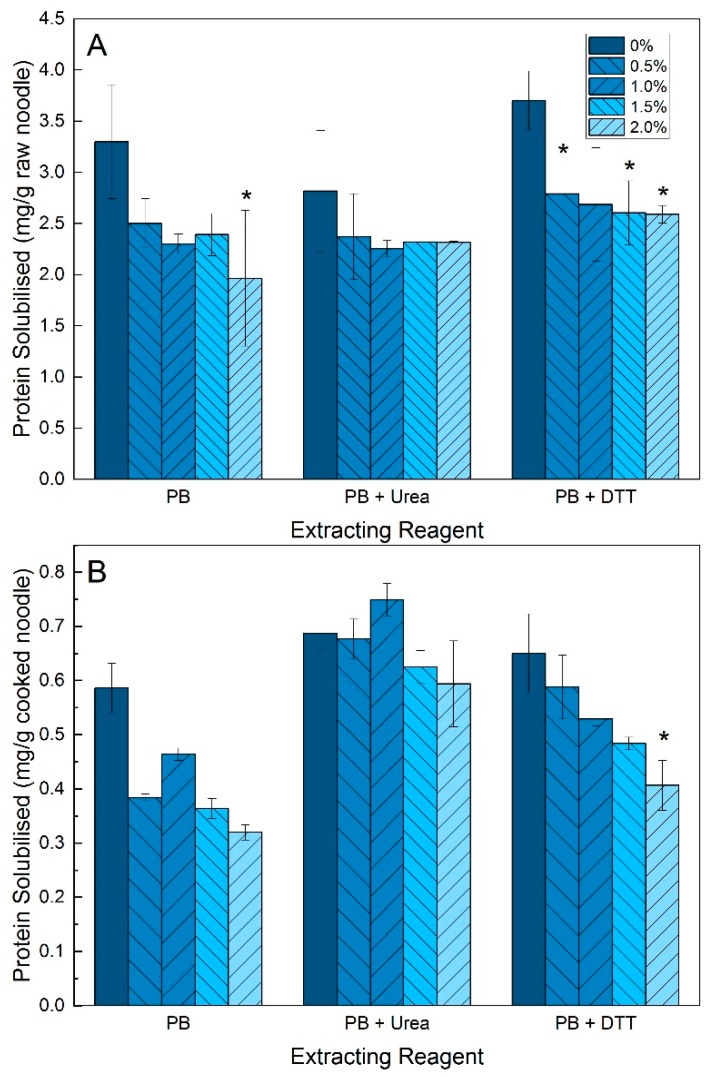
Protein solubility of (**A**) raw and (**B**) cooked noodles with 0%, 0.5%, 1.0%, 1.5% and 2.0% transglutaminase in extracting reagents phosphate buffer (PB; 0.1 M; pH 6.8), 8 M urea in PB (PB + Urea), 0.05 M dithiothreitol in PB (PB + DTT) and 0.05 M sodium dodecyl sulphate in PB (PB + SDS); * indicates significant difference (*p* < 0.05) compared to control (0% TG) in the same extracting reagent group; error bars represent S.D. based on duplicates.

For raw noodles (Figure 2A) in PB, the control noodles without TG treatment had the highest protein solubilised. In PB + urea, the differences between control and TG-treated noodles across all concentrations were not significantly different, although the amount of protein solubilised was slightly higher in the control. In PB + DTT, the amount of protein solubilized in the control noodles was significantly higher than all other samples, although there were no apparent concentration effects amongst the TG-treated noodles. For cooked noodles (Figure 2B) in PB, the protein solubility was similarly the highest in the control noodles. In PB + urea, there were again no differences between the control and other noodle samples. In PB + DTT, the amount of protein solubilized in the control noodles was significantly higher than all other samples, with a decreasing trend of solubilized protein with increasing TG concentration. Comparing raw and cooked noodles, cooked noodles had higher protein solubility in additional urea and DTT reagents than in PB alone. More protein was also solubilised in the raw noodles compared to the cooked noodles, even after accounting for water absorption during cooking.

### 3.3. Thermal Properties (DSC)

Thermal transitions (peaks) were observed in the region from 55 to 75 °C, which corresponded to pure wheat starch gelatinisation (T_o_: 58.0 °C; T_p_: 63.4 °C; T_e_: 70.6 °C; ΔH: 12.7 J/g). There were no significant differences (*p* > 0.05) between samples for all parameters and TG concentrations (Table 1). However, a trend of increasing onset temperature and gelatinisation enthalpy with increasing TG concentration was observed.

### 3.4. Textural Properties

**Figure 3:**
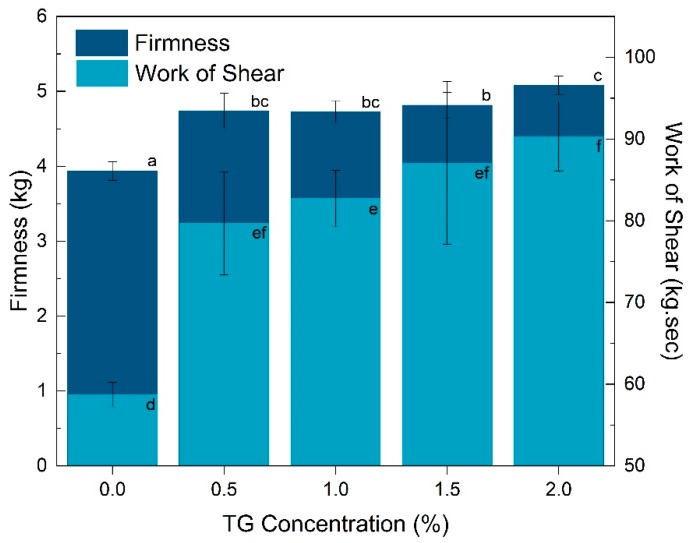
Bulk textural properties firmness (kg) and work of shear (kg·sec) of noodles at different transglutaminase (TG) concentrations; different letters represent significant difference (*p* < 0.05) between samples.

The control noodles (0% TG) were the lowest in firmness and work of shear, and were significantly different (*p* < 0.05) from noodles treated with 0.5–2% TG for both parameters (Figure 3). Noodles with 2% TG were the highest in firmness and work of shear compared to noodles with 0.5–1.5% TG (non-significant). A trend of increasing firmness and work of shear with increasing TG concentration was observed. 

### 3.5. Water Absorption

Water absorption rates of noodles treated with 0 to 2% transglutaminase (TG) are summarised in Table 2. There were no significant differences in the rate of water absorption amongst all noodle samples with and without transglutaminase. 

### 3.6. In Vitro Digestion (Glucose Release)

**Figure 4:**
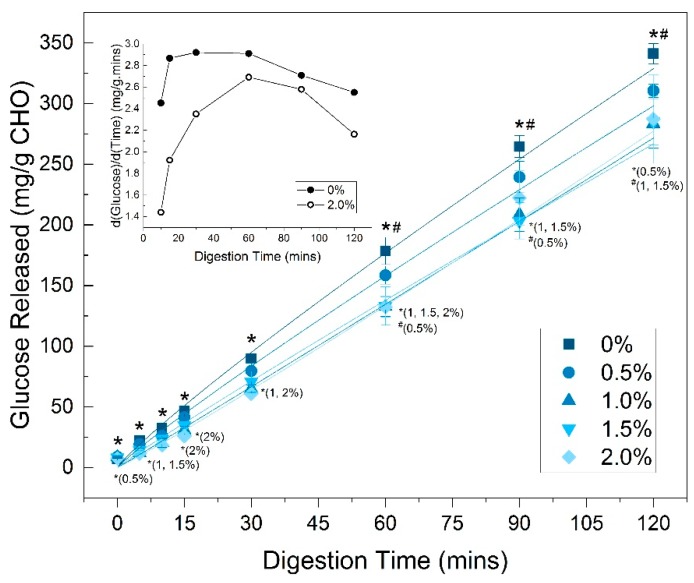
Glucose released (mg/g carbohydrate) in cooked noodles with 0%, 0.5%, 1.0%, 1.5% and 2.0% transglutaminase from 0 to 120 min of in vitro intestinal phase digestion; error bars represent S.D. based on duplicates; * represents significant difference from control at a time point (*p* < 0.05), # represents trending difference from control at each time point (*p* < 0.10); inset: first-order derivate (slope) of glucose release with digestion time (i.e., rate of glucose release) for noodles with 0% (●) and 2.0% (◯) transglutaminase.

The amount of glucose released per gram of carbohydrate in cooked noodles during intestinal phase digestion over 120 min is shown on Figure 4. The carbohydrate content was calculated based on the water absorption rates (Table 2) and composition of wheat flour. All noodle samples showed an increase in the amount of glucose released with time, which corresponds to the digestion of starch in noodles to glucose by α-amylase and amyloglucosidase enzymes with time. There were significant (*p* < 0.05) or trending (*p* < 0.10) differences in the amount of glucose released for a TG-treated noodle compared to the control (0% TG) noodle at all time points. The difference in the amount of glucose released between the control (0% TG) and TG noodle samples widened as the digestion proceeded. For example, at 15 min of digestion, the amount of glucose released ranged from 18.4 (2.0% TG) to 32.4 mg/g carbohydrate (0% TG), while at 120 min of digestion, the difference ranged from 287 (2.0% TG) to 341 mg/g carbohydrate (0% TG). At 120 min, the reduction in the amount of glucose released from the control were 9.0%, 16.9%, 16.5% and 15.8% for 0.5%, 1%, 1.5% and 2.0% TG, respectively. Overall, there were distinct differences between the control (0% TG) and TG-treated (0.5–2% TG) noodles, especially from 30 min of digestion onwards. However, the amounts of glucose released with time were relatively close between the 1%, 1.5% and 2.0% TG noodle samples. 

The inset in Figure 4 shows the rate of glucose released with digestion time (first-order derivative) for the control (0% TG) and 2% TG noodle samples. The control (0% TG) sample had a higher rate of glucose release than the noodles with 2% TG across all digestion time points. For the control (0% TG) sample, the rate of glucose release started higher than the 2% TG noodle sample, reached a plateau during 15 to 60 min of digestion, and then started to decrease. The rate of glucose release for the 2% TG noodle sample, however, slowly increased from 15 to 60 min of digestion, peaked at the 60 min time point, and then decreased. 

## 4. Discussion and Conclusions

The objectives of the study were to determine if transglutaminase enzyme could reduce starch hydrolysis in Asian wheat noodles via increased protein network-binding. The hypothesis was that transglutaminase enzyme would form covalent crosslinks within the protein (gluten) matrix, which serves as a barrier around the starch granules against enzymatic access to reduce the rate and/or extent of starch hydrolysis. A higher transglutaminase concentration would correspondingly form more crosslinks, which would strengthen the protein barrier, resulting in greater reduction in starch digestibility. 

Confocal microscopy showed visible changes in the appearance of the protein and starch matrix with TG concentration. Without transglutaminase, starch granules appeared to be in a laminar arrangement with a thin protein matrix distributed alongside. This is likely due to the alignment of the starch granules in the direction of noodle extrusion [25]. With transglutaminase, post-extrusion crosslinking lost the starch orientation, and also rearranged the protein matrix surrounding the starch granules. Similar microstructures were also found in yellow alkaline noodle with transglutaminase treatment using scanning electron microscopy (SEM), and it was also shown that sheeting the dough after transglutaminase crosslinking has taken place strongly orientates the starch granules [26]. This demonstrates the tight embedment of starch granules within the protein matrix, and how it may act as a barrier to enzymatic digestion. 

The integrity and strength of the protein network was compared based on protein solubility, (starch) thermal and bulk properties. There was a general trend of decreased protein solubility across all extracting reagents in noodles with TG-treatment compared to noodles without TG, indicating an overall greater degree of protein network-binding, which restricts protein solubilisation [27]. However, concentration effects were not as apparent, suggesting a plateau in the degree of crosslinking achievable by TG at the concentrations tested. The changes to protein solubility in PB with urea, which disrupts non-covalent hydrogen bonds, was the smallest with TG-treatment for both cooked and raw noodles. This indicates that TG does not promote additional hydrogen bonding within the protein network after crosslinking. DTT, on the other hand, disrupts and reduces the covalent disulphide (S-S) bonds in gluten [28]. However, not all S-S bonds in the protein aggregates would be accessible and reduced by DTT without the presence of a chaotropic agent, such as urea. Based on this observation of decreasing protein solubility in PB with DTT for TG-treated noodles, it can only be inferred that the crosslinked G-L bonds may serve as a barrier to free soluble proteins entrapped within the protein network held by some of the S-S linkages. Cooking is a process which results in the denaturation and polymerization of glutenin and gliadin via disulphide linkages and hydrogen bonds [29,30,31,32]. The solubility in PB with urea or DTT is therefore higher for cooked noodles compared to only using PB, as the denatured protein fractions are more susceptible to disruption via these reagents. 

The presence of a protein network surrounding the starch granules can also alter the gelatinisation behaviour of starch in terms of its thermal properties. A small increase (non-significant) in onset temperature as well as gelatinisation enthalpy with transglutaminase concentration was observed. A higher onset temperature may have been due to impeded water absorption into the denser protein network, thus requiring a higher temperature for gelatinisation [33]. An increase in gelatinisation enthalpy indicates that more energy was required to ‘melt’ the starch, as a result of the denser protein network acting as an effective barrier to gelatinisation. The same phenomenon was observed in pastas or noodles with added protein [16,34,35]. However, these observed effects were relatively small and non-significant in terms of thermal properties, and some studies showed that changes to the protein network do not affect gelatinization enthalpy, since the starch structure itself is not altered [36]. Results should therefore be interpreted with caution. 

Transglutaminase crosslinking at the microstructural level also manifested in properties at the bulk level. Firmness and work of shear increased with transglutaminase concentration, correlating with the strength of the protein network resisting deformation. The increase in textural strength with transglutaminase treatment is consistent with other noodle and pasta systems [37,38,39]. For water absorption, however, several studies have shown that transglutaminase treatment reduces water uptake, due to a tighter network between protein and starch [39,40]. This was not observed in this study, which may be due to differences in cooking parameters, for example, whether noodles were cooked with an optimal cooking time or a fixed duration, and the subsequent extent of starch gelatinisation and protein denaturation. 

In vitro digestion results showed that the amount of glucose released was significantly lower for TG-treated noodle samples across 120 min of digestion. Increasing TG concentration further limited glucose release, although the reductions were similar at 1%, 1.5% and 2.0%. Therefore, TG activity can decrease starch digestibility, but concentration effects may plateau, especially at high TG concentrations. The rate of glucose release was also higher for the control during the first 60 min of digestion compared to TG-treated noodles (2% TG). Starch hydrolysis is a two-step process, starting first with enzymatic (α-amylase/α-glucosidase) access to the substrate (starch), followed by starch catalysis into glucose [41,42]. The slower rate of glucose release in TG-treated noodles during the first 60 min of intestinal phase digestion suggests that the initial enzyme attachment to starch was impeded, but subsequent rates of starch hydrolysis to glucose were similar to the control once the enzymes gained access to starch. These results support the hypothesis that the strengthened protein network surrounding the starch granules, which limits enzymatic access, is responsible for the reduction in digestibility.

Overall, there is evidence to support that in vitro starch digestibility can be reduced with transglutaminase crosslinking, and is likely to be attributed to the effect on the protein network. Limiting enzymatic access in the first stage of starch hydrolysis with a dense protein network would be more effective than strategies which slow down starch hydrolysis in the second stage, as enzymatic access is the rate-limiting step [41,42,43]. Using as low as 0.5% transglutaminase provided an appreciable reduction in glucose release and protein network strengthening (based on firmness), while further increasing the TG concentration did not necessarily reduce starch digestibility proportionally. Limited protein (i.e., glutamine and lysine residuals) may be available for crosslinking, and further increasing the protein content of noodles (e.g., egg noodles) may see a larger effect with transglutaminase, as observed in gluten-free pasta made with high protein legume flours [37,40]. Protein-binding using transglutaminase is an effective method for providing a barrier from enzymatic access while, at the same time, improving textural qualities [39]. The use of hydrocolloids, for example, may be able to reduce starch digestibility but often compromises on noodle texture [44]. This study was limited in that the effects of digestion on the protein network were not characterised, and in regard to how the changes in protein network may have affected starch digestibility in parallel. In vitro digestion results may also not be reflective of actual glycaemic response in human subjects. Future work should include these aspects, as well as quantitative characterization of the protein network [45]. 

## Figures and Tables

**Table 1:** Onset, peak and end temperature (°C) and gelatinisation enthalpy (J/g) (±S.D.) of noodles at different transglutaminase (TG) concentrations (%).

TG Concentration (%)	Onset Temperature, T_o_ (°C)	Peak Temperature, T_p_ (°C)	End Temperature, T_e_ (°C)	Enthalpy of Gelatinisation, ΔH (J/g)
0	57.1 ± 0.2	65.7 ± 0.9	72.4 ± 1.0	3.4 ± 0.2
0.5	56.7 ± 0.4	65.9 ± 0.2	72.8 ± 0.6	4.8 ± 0.7
1.0	58.6 ± 3.6	66.3 ± 1.6	73.6 ± 1.5	5.4 ± 1.1
1.5	58.1 ± 0.9	65.4 ± 0.1	72.7 ± 9.2	4.9 ± 0.9
2.0	60.0 ± 2.1	66.0 ± 1.1	73.5 ± 0.6	4.8 ± 0.6

**Table 2:** Water absorption (%) (±S.D.) rates of noodles at different transglutaminase (TG) concentrations (%) with cooking.

TG Concentration (%)	Water Absorption (%)
0	87.3 ± 9.4
0.5	93.8 ± 2.7
1.0	90.0 ± 1.7
1.5	89.7 ± 4.6
2.0	91.1 ± 2.0
